# The Role of Mucins in Esophageal Inflammatory Diseases

**DOI:** 10.3390/jpm16020093

**Published:** 2026-02-05

**Authors:** Laura Arias-González, Alfredo J. Lucendo

**Affiliations:** 1 Department of Gastroenterology, Hospital General de Tomelloso, Vereda de Socuéllamos s/n, 13700 Tomelloso, Spain; ajlucendo@hotmail.com; 2 Centro de Investigación Biomédica en Red de Enfermedades Hepáticas y Digestivas (CIBERehd), Instituto de Salud Carlos III, 28029 Madrid, Spain; 3 Instituto de Investigación Sanitaria Princesa, 28006 Madrid, Spain

**Keywords:** inflammatory disorders, epithelial barrier, mucosal immunology, esophagus, mucins, gastroesophageal reflux disease (GERD), eosinophilic esophagitis (EoE), Barrett’s esophagus, esophageal cancer

## Abstract

Mucins are high-molecular-weight glycoproteins that form the main structural component of the mucus covering epithelial surfaces in the gastrointestinal, respiratory, and urogenital tracts. They support epithelial integrity by protecting against microbial invasion, dehydration, and mechanical or chemical insults, while facilitating the transit of luminal contents. Beyond their structural function, mucins play key roles in molecular recognition. Their extensive glycosylation enables interactions with a wide range of molecules and allows the discrimination between pathogenic and commensal microorganisms at mucosal surfaces. Mucins help maintain mucosal homeostasis by preventing pathogen adhesion and colonization, while simultaneously providing nutrients to commensal species, supporting their stability, and maintaining spatial segregation from epithelial surfaces. Aberrant expression of mucin subtypes or alterations in their glycosylation patterns are associated with numerous diseases, including a wide spectrum of cancers and inflammatory disorders. The immunological relevance of the esophageal mucosa has only recently been recognized. Advances in the study of the esophageal mucosa-associated immune surveillance system and its interactions with structural components of this organ’s surface, including mucins, have shed light on unique pathological processes in the esophagus, such as Barrett’s esophagus, gastroesophageal reflux disease, and eosinophilic esophagitis. This review focuses on the role of esophageal mucins in inflammation, compiling current evidence to provide an integrated overview of mucin-driven inflammatory mechanisms.

## 1. Molecular and Functional Aspects of Mucins

Mucins are the primary components of the mucus that lines the mucosal epithelia of the gastrointestinal tract. They are glycoproteins belonging to a family of proteins characterized by serine-, threonine-, and proline-rich (STP) sequences or tandem-repeat domains in which serine and threonine residues undergo extensive O-glycosylation, giving rise to a distinctive “bottle-brush” conformation. Genetic polymorphisms in the number of STP repeats have been reported among individuals [1]. Approximately 80% of mucin mass consists of glycans, which form a dense glycan-rich shield that protects the protein backbone from degradation by endogenous or microbial proteases [2]. Additional post-translational modifications, such as sialylation and sulphation, may also occur in mature mucins [3]. Glycosylation patterns vary between individuals and among different gastrointestinal regions, depending on the expression of specific glycosyltransferases. However, in healthy mucosa, a given region typically maintains a stable glycosylation profile; factors such as microbial interactions or low-fiber diets can disrupt this pattern and alter mucin conformation [4].

**Figure 1:**
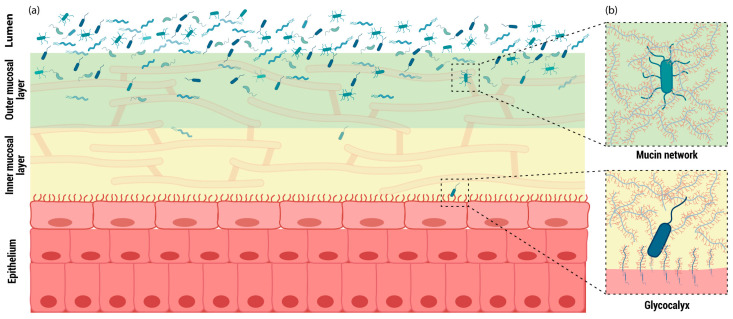
General structure of the gastrointestinal tract mucosa. (**a**) Distribution of mucosal layers in the gastrointestinal tract; (**b**) Interaction between bacterial proteins and mucins within the mucin network, and membrane-bound mucins associated with the apical epithelium.

Mucins are classified into two structural types: membrane-bound and secreted mucins. Membrane-bound mucins are synthesized and anchored to the epithelial surface through a transmembrane domain, whereas secreted mucins are released into the mucus layer to form extensive multimeric networks (Figure 1). Membrane-bound mucins play essential roles in recognizing commensal and pathogenic bacteria, contributing to epithelial protection and mediating homeostatic responses in the inner mucus layer through intracellular signal transduction or interactions with the cytoskeleton [5]. These signaling pathways regulate key cellular processes, including growth, motility, differentiation, and inflammation [3] (Figure 1).

Membrane-bound mucins contribute to epithelial protection by linking apical surface sensing to intracellular signaling pathways that regulate cytoskeletal organization and junctional stability. For example, MUC1 interacts with β-catenin in a phosphorylation-dependent manner, supporting adherens junction integrity, while MUC4 modulates protective signaling through ErbB2 activation. Together, these mechanisms help maintain epithelial integrity in response to stress or injury.

In contrast, secreted mucins play a dual role in the mucus layer, balancing microbial colonization with epithelial protection through spatial and biochemical organization. As major structural components of the outer mucus layer, they form highly glycosylated networks that provide attachment sites and nutrient sources for commensal microorganisms, thereby facilitating controlled microbial colonization. At the same time, this mucin-rich matrix maintains most bacteria at a physical distance from the epithelial surface, limiting direct epithelial contact and preventing pathogenic invasion. This compartmentalization allows secreted mucins to support a stable microbial community while preserving epithelial barrier integrity.

Among the membrane-bound mucins described in the gastrointestinal tract are MUC1, MUC3A, MUC3B, MUC4, MUC12, MUC13, MUC15, MUC16, MUC17, MUC20, MUC21, and MUC22. Secreted mucins include MUC2, MUC5B, MUC5AC, MUC6, and MUC7, although only MUC2, MUC5AC, MUC5B, and MUC6 form the gel-like mucus matrix [6].

In summary, the mucus layer is composed predominantly of mucins, which protect against fluid shear stress, mechanical and chemical injury, and bacterial invasion, while supporting the maintenance of a stable microbiota throughout the gastrointestinal tract. Although its composition varies along the tract, the mucus layer generally consists of an inner layer in direct contact with the apical epithelium and an outer layer formed by a mucin gel.

## 2. Role of Mucins in Inflammatory Disorders

The well-established role of mucins in cellular recognition within mucosal epithelia highlights their importance in distinguishing pathogenic from commensal microorganisms. This capacity is essential for maintaining mucosal homeostasis while allowing the immune system to mount appropriate responses to infection. These properties make mucins compelling candidates for investigating inflammatory processes across different organs.

Although in vitro studies, animal models, and some clinical observations support an immunomodulatory role for mucins, the specific signaling pathways involved remain largely undefined. The main findings in this regard are described below:

### 2.1. Glycosylation Changes as Markers of Inflammation

Pro-inflammatory cytokines such as IFN-γ, IL-1, and TNF-α can modify the glycosylation of MUC1, MUC5, and MUC16 [7], while MUC7 exhibits glycosylation alterations in recurrent aphthous stomatitis, pyelonephritis, and rheumatoid arthritis [8,9,10]. These findings underscore aberrant glycosylation as a hallmark of mucosal inflammation.

### 2.2. Cytokine-Driven Regulation of Mucin Expression

Inflammatory cues reshape mucin expression profiles across tissues. MUC1 is upregulated by inflammatory stimuli in epithelial cells of the ovary, eye, breast, and oral mucosa [11,12,13,14], whereas MUC2 expression is reduced in IBD [15], and MUC4 is increased in eosinophilic esophagitis and Barrett’s esophagus [16,17]. Additional inflammation-associated changes include altered levels of MUC6 in Crohn’s disease, MUC7 in oral, gastrointestinal, urinary tract, and joint inflammation [8,18], MUC13 in bacterial infection [19], and MUC15 and MUC22 in response to viral stimuli [20,21].

### 2.3. Immunomodulatory Activity of Mucins 

Beyond their expression patterns, several mucins actively participate in immune regulation. MUC1 modulates T-cell and dendritic-cell responses by attenuating TLR signaling and cytokine production [22,23], while its tumor-associated form contributes to immune evasion [13]. MUC5B binds SIGLEC-8 on eosinophils and promotes TLR4-mediated immune recruitment in salivary glands [24,25].

### 2.4. Transcriptional and Epigenetic Regulation

Mucin transcription is also responsive to inflammatory signals. The MUC2 promoter contains an NF-κB binding site, relevant to IBD pathogenesis [15]. MUC3 polymorphisms have been associated with intestinal inflammation [19]. Various inflammatory stimuli—including IL-1β, IL-6, TNF-α, IL-4, IL-13, IL-9, and bacterial LPS—upregulate mucins such as MUC2, MUC5AC, MUC5B, and MUC6 [19], while epigenetic mechanisms additionally regulate MUC5B expression [26]. MUC16 expression is enhanced by IL-1, TNF-α, and LPS in inflammatory cardiac conditions [27], and rare genetic variants have been linked to eosinophilic esophagitis [28].

### 2.5. Mucins in Cancer-Related Inflammation

In cancer, MUC1 is induced via NF-κB and STAT1 pathways [29], as well as through STAT3 activation downstream of EGF and IL-6 [29,30]. Increased MUC1 expression has been documented across multiple epithelia during inflammation [11,12,14,31,32].

Collectively, alterations in glycosylation, expression levels, and genetic variants link mucins closely to inflammation-associated pathways, including immune-cell recruitment, cytokine-mediated regulation, Toll-like receptor signaling, and interactions with inflammation-responsive transcription factors. These features highlight the potential of mucins both as modulators and indicators of inflammatory responses.

## 3. Mucins in Esophageal Inflammation

### 3.1. The Esophageal Mucosa

Mucosal barriers represent the first line of defense against the external environment, while simultaneously permitting bidirectional exchange and molecular crosstalk between the epithelium and the lumen, processes that are critical for the maintenance of gastrointestinal homeostasis. The esophagus functions to transport ingested material from the oral cavity to the stomach, and its mucosal lining provides protection against mechanical stress associated with swallowing, as well as against exposure to luminal allergens and microbial pathogens.

The anatomical organization of the esophagus is comparable to that of the distal segments of the gastrointestinal tract and comprises, from the outside inward, a muscular layer, submucosa, muscularis mucosae, lamina propria, and epithelium. Unlike other digestive organs, the extraperitoneal location of the esophagus means that this organ lacks a serosal layer in most of its length, which is replaced by an adventitia of loose connective tissue. This fact has critical clinical and pathophysiological consequences for fibrosis and healing processes [33]: the lack of an external elastic serosa can facilitate the spread of inflammatory and fibrotic processes from the esophagus to adjacent structures in the mediastinum, leading to a reduction in the diameter of the organ’s lumen, as observed in chronic gastroesophageal reflux or eosinophilic esophagitis (EoE) [34]. However, unlike lower regions of the digestive tract, the esophageal epithelium consists of a stratified squamous mucinous epithelium, resembling that of the skin. Epithelial cell renewal occurs within the basal layer, where proliferating cells displace older cells toward the luminal surface as they progressively lose contact with the basement membrane. In the basal compartment, cells exhibit a columnar morphology with round nuclei; during differentiation and upward migration, they gradually flatten and become densely packed [35].

As in the remaining organs of the digestive tract, there is a protective mucosal layer above the outermost epithelial layer. However, in contrast to other gastrointestinal epithelia, the esophageal epithelium lacks goblet cells under physiological conditions. Accordingly, mucins in the esophagus are secreted either by submucosal glands or by non-specialized epithelial cells lining the mucosa.

In the normal esophageal epithelium, MUC1 and MUC4 are the mucins predominantly expressed, although MUC5B is also commonly detected. In addition, MUC20, MUC21, and MUC22 have also been found. Among these, MUC1, MUC4, MUC20, MUC21, and MUC22 are classified as membrane-bound mucins, which is consistent with the fact the squamous esophageal epithelium is a not secretor of mucus. In contrast, MUC5B is synthesized in submucosal glands and secreted into the mucus layer [36,37].

Regarding their spatial distribution within the epithelium, MUC1 and MUC4 are primarily expressed in the superficial layer, where the most differentiated squamous cells reside. This localization suggests a role in epithelial surface protection and cellular signaling, particularly through the modulation of cell–cell interactions. In contrast, the expression of MUC5B in the submucosal glands indicates a more classical function related to luminal lubrication and mechanical protection [35].

### 3.2. Esophageal Mucins

The expression of mucins in the esophagus varies under health and disease conditions. On the one hand, MUC1, MUC4, and MUC5B are typically found in the healthy esophageal epithelium, while MUC2, MUC3, MUC5AC, MUC6, MUC12, MUC13, MUC17 and MUC19 are predominantly detected in specific disease contexts, such as metaplasia or chronic inflammation. Although data remain limited, the esophagus have been found to express MUC15, MUC16, MUC20, MUC21, and MUC22 in certain condition. In addition, salivary mucins, including MUC5B, MUC7 and MUC19 may also be present in the esophagus as they are swallowed from the oral cavity and will therefore be addressed in this review.

All aforementioned mucins and associated findings related to inflammatory processes are summarized in Table 1.

## 4. Inflammatory Esophageal Diseases

**Figure 2:**
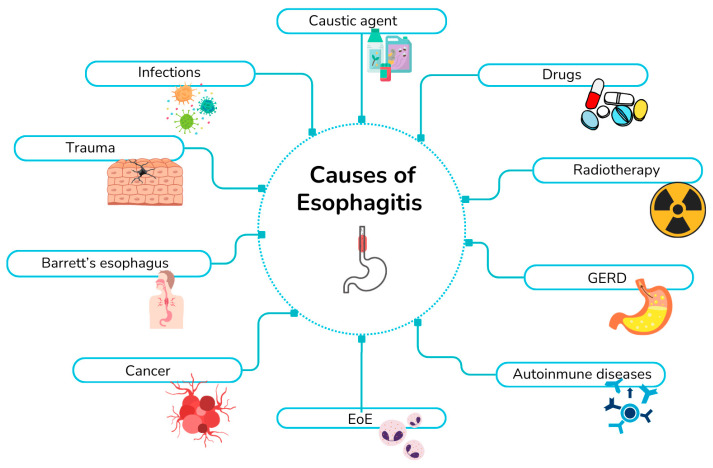
Schematic overview of esophagitis etiologies.

Esophagitis may arise from multiple etiologies (Figure 2). In this section, we review the major inflammatory conditions affecting the esophagus and examine current evidence regarding the involvement of mucins in these processes. The causes of esophagitis include gastroesophageal reflux disease (GERD), EoE, infectious esophagitis, Barrett’s esophagus, esophageal adenocarcinoma, autoimmune disorders, as well as inflammation induced by medications, radiation, caustic agents, and mechanical trauma.

### 4.1. Gastroesophageal Reflux Disease

Gastroesophageal reflux disease (GERD) is characterized by the retrograde flow of gastric and/or duodenal contents into the esophagus, resulting in symptoms such as heartburn, regurgitation and retrosternal pain. Prolonged exposure of the esophageal mucosa to acidic refluxate can lead to complications including peptic esophagitis, reflux hypersensitivity, Barrett’s esophagus, and non-erosive reflux disease (NERD) [38].

In vitro models of the human esophagus have demonstrated that sustained acid exposure significantly increases MUC1 expression [39], supporting the association between MUC1 overexpression and its function as a stress-response molecule in epithelial cells. Similar roles for MUC1 have been reported in other esophageal conditions, including Barrett’s esophagus and its progression to adenocarcinoma [40].

MUC5AC is not detected in the normal esophageal epithelium but is upregulated in GERD. Conjugated bile acids present in refluxate have been shown to mediate this increased expression [41]. In addition, MUC5AC—together with other secreted mucins such as MUC6 and MUC2—can be induced by hydrochloric acid or pepsin, despite their absence from the normal esophageal mucosa under physiological conditions. Reflux-associated stimuli may therefore promote MUC5AC expression as an early protective response during the initial stages of GERD [42].

As discussed previously, some mucins detected in the esophagus originate from saliva secreted by the oral mucosa. Salivary mucins, along with other salivary components, play a critical role in buffering acidic refluxate within the esophageal lumen. In patients with GERD, salivary mucin function is impaired [43], and reduced esophageal mucin levels have been correlated with increased severity of reflux esophagitis [44], further supporting their protective role.

Notably, the severity of reflux-induced injury influences both mucin production and the reversibility of these alterations. In erosive reflux disease, esophageal mucin production is reduced compared with healthy controls. This reduction appears to be reversible in mild cases but becomes irreversible in more severe disease, even following mucosal healing [44]. Accordingly, MUC1 and MUC4 expression levels are lower in patients with erosive esophagitis than in those with NERD or in healthy controls. In contrast, MUC3 and MUC5AC expression is increased in erosive esophagitis [42]. These differential patterns may reflect functional differences among mucin subtypes, as membrane-bound mucins (e.g., MUC1 and MUC4) are primarily involved in signal transduction and environmental sensing, whereas secreted mucins mainly contribute to mucus gel formation and epithelial protection. Overall, mucin expression profiles in patients with NERD more closely resemble those of healthy individuals than those observed in erosive esophagitis.

Among current therapeutic strategies for GERD, proton pump inhibitors (PPIs) are of particular relevance. In addition to their established effects on gastric acid and pepsin suppression, PPIs have been shown to enhance gastric mucin secretion [45].

MUC3 expression in the esophageal mucosa of patients with erosive GERD has been reported to be higher than in individuals without reflux and in those with non-erosive disease, although these differences did not reach statistical significance. At the transcript level, a significant correlation between IL-6 and MUC3 expression was observed in both control and erosive reflux groups, suggesting that mucin expression in the esophageal mucosa may be modulated by the acidity and chemical composition of gastroesophageal refluxate [42]. Persistent exposure to acidic gastric contents promotes inflammatory responses in GERD. In this context, MUC1 has been shown to inhibit Toll-like receptor (TLR) activation in epithelial cells, thereby reducing the production of pro-inflammatory mediators such as IL-8, IL-1β, and TNF-α. These cytokines, in turn, negatively regulate TLR signaling while simultaneously enhancing MUC1 expression, suggesting the existence of a feedback mechanism linking mucin expression and inflammatory signaling pathways [46].

### 4.2. Barrett’s Esophagus and Esophageal Carcinoma

Although Barrett’s esophagus and its progression to esophageal adenocarcinoma are distinct pathological entities, they are discussed together here to provide a dynamic view of disease evolution from a premalignant to a malignant state, particularly with respect to alterations in esophageal mucin expression and regulation. Both conditions are associated with chronic inflammatory processes; however, they are not classified as primary inflammatory diseases, as inflammation is not their defining pathological feature. Barrett’s esophagus is widely regarded as a complication arising from the chronic impact of GERD on the esophageal mucosa. It represents a premalignant condition characterized by the metaplastic replacement of the normal stratified squamous epithelium with a columnar, intestinal-like phenotype, which may subsequently progress to esophageal adenocarcinoma. As a consequence of this cellular reprogramming, mucus-secreting cells emerge and express mucins such as MUC1, MUC2, MUC3, MUC5AC, and MUC6. Reflux-induced metaplasia promotes a pro-inflammatory microenvironment, enhancing oxidative DNA damage through mediators including TNF-α, prostaglandin E2 (PGE2), and NF-κB signaling pathways [47].

A meta-analysis by Niv et al. [48] demonstrated that mucin expression follows a gradient along the continuum from premalignant to malignant esophageal lesions. Specifically, mucin expression levels were lowest in Barrett’s mucosa with low-grade dysplasia (LGD), increased in high-grade dysplasia (HGD), and highest in esophageal adenocarcinoma. Expression of MUC2, MUC3, MUC5AC, and MUC6 was significantly higher in adenocarcinoma than in HGD, and higher in HGD than in LGD. In contrast, MUC1 and MUC4 exhibited an inverse pattern, with higher expression in LGD compared with HGD and adenocarcinoma [48]. These findings highlight a pronounced upregulation of mucins not normally expressed in the healthy esophagus, alongside a downregulation of mucins typically present under physiological conditions.

However, divergent observations have been reported, particularly with respect to MUC3 expression. Some studies have shown that MUC3 is highly expressed in non-dysplastic Barrett’s esophagus but progressively decreases during dysplastic progression—from intestinal metaplasia to indefinite dysplasia, LGD, and HGD—with complete loss observed in cases of high-grade dysplasia [49]. Other investigations have failed to confirm this trend, although they consistently report an association between mucin dysregulation and inflammatory signaling. These discrepancies likely reflect differences in methodological approaches, including antibody specificity and tissue sampling strategies, as well as biological factors such as stage-dependent and context-specific regulation of MUC3 during disease progression.

Inflammatory mediators such as IL-6 and TNF-α activate transcription factors including NF-κB, STAT1, and STAT3 in gastrointestinal epithelial cells, thereby driving changes in mucin gene expression. MUC1 is associated with enhanced epithelial cell proliferation and survival, whereas MUC2 normally contributes to limiting inflammation at the mucosal surface; both are frequently downregulated in chronic inflammatory settings, including those associated with environmental exposures such as smoking and alcohol consumption. These pro-inflammatory factors induce epigenetic modifications that facilitate molecular alterations and promote the accumulation of genetic mutations in epithelial cells, thereby creating a permissive environment for malignant transformation. Mucin genes such as MUC2, MUC5AC, and MUC6 appear particularly susceptible to these regulatory disturbances, increasing the risk of neoplastic progression. Conversely, membrane-bound mucins, including MUC1 and MUC4, are often upregulated in Barrett’s esophagus and esophageal adenocarcinoma, whereas secreted mucins such as MUC2 tend to decline during neoplastic progression, resulting in compromised mucosal barrier function and impaired epithelial repair mechanisms [50].

Beyond their potential utility as diagnostic and prognostic biomarkers, these findings underscore a mechanistic link between inflammatory signaling and alterations in mucosal composition. Although controversy persists regarding the expression patterns of individual mucin subtypes, it is evident that inflammatory stimuli profoundly alter mucin production in the esophagus compared with healthy tissue. In this context, MUC6—normally absent from the esophageal epithelium but expressed in other regions of the gastrointestinal tract—has been associated with intestinal inflammatory responses and chronic gastritis related to *Helicobacter pylori* infection. In both settings, NF-κB pathway activation appears to mediate mucin dysregulation [51].

Collectively, these studies demonstrate aberrant mucin expression patterns in Barrett’s esophagus and esophageal adenocarcinoma, although further research is required to clarify the precise mechanisms governing these alterations.

### 4.3. Eosinophilic Esophagitis

EoE is a chronic, immune-mediated inflammatory disorder of the esophagus and has emerged as the second most common cause of chronic esophagitis after GERD. It is currently the leading cause of dysphagia and food impaction in children and young adults [52]. Several studies have investigated mucin expression patterns in the esophageal mucosa of patients with EoE. Among the mucin gene family, MUC4 has been identified as the only mucin significantly upregulated in active EoE compared with control samples [53]. Notably, MUC4 overexpression has also been reported in Barrett’s esophagus and high-grade intraepithelial neoplasia [54], potentially reflecting enhanced epithelial differentiation or remodeling. Importantly, effective anti-inflammatory treatment in EoE has been shown to restore MUC4 expression to baseline levels, both at the transcriptional [16] and protein levels [55]. In parallel with MUC4 upregulation, MUC1 and MUC5B are significantly downregulated in active EoE [16], as are MUC21 and MUC22 genes [53], indicating that mucin dysregulation in EoE is broad rather than limited to a single mucin subtype. In addition, suprabasal staining of MUC21 has been proposed as a marker of epithelial differentiation in squamous cell carcinoma [37], suggesting its potential utility as an indicator of esophageal epithelial differentiation in other pathological contexts.

Despite the limited number of studies available, consistent findings indicate that active EoE is characterized by decreased expression of MUC1 and MUC5B and increased expression of MUC4, reflecting a disease-associated shift in mucin expression. These changes are accompanied by activation of inflammatory signaling pathways, including MyD88 and NF-κB, increased expression of cytokines such as IL-1β, IL-6, IL-8, and IL-10, and upregulation of immune effector molecules including PER-1, iNOS, and GZMA. Furthermore, components of the NKG2D axis (KLRK1, IL-15, MICB) are elevated, suggesting a coordinated inflammatory response linked to mucin dysregulation. Importantly, these molecular alterations are reversed following the six-food elimination diet (SFED) and subsequent mucosal healing [16].

The interaction between mucus and eosinophils appears to be bidirectional. Eosinophil-driven mechanisms promote mucus hypersecretion and alter its biophysical properties, while changes in mucus composition may, in turn, exacerbate eosinophilic inflammation. This reciprocal relationship has been well characterized in asthma, where a predominance of MUC5AC over MUC5B correlates with increased eosinophil infiltration, potentially implicating MUC5AC in eosinophil survival, possibly through reduced eosinophil apoptosis [56]. A similar regulatory mechanism may operate in EoE, in which altered mucus composition and epithelial remodeling could influence eosinophil persistence and activation, although this hypothesis remains to be fully elucidated.

More recently, MUC16 has been identified as a candidate gene in familial EoE, with evidence of shared rare variants among affected relatives and differential gene expression. These findings suggest a potential role for MUC16 in genetic susceptibility and epithelial barrier dysfunction in EoE [28].

In summary, although mucin expression in the esophageal mucosa has been described, the functional role of mucins in EoE-associated inflammation remains poorly understood. It is well established that mucin expression varies according to epithelial differentiation status across esophageal layers. While mucin dysregulation appears to be a hallmark of EoE-inflamed mucosa, its significance beyond serving as a potential biomarker has yet to be fully defined.

### 4.4. Infectious Esophagitis

Esophageal infections are uncommon in the general population but occur more frequently in individuals with compromised immune function, including patients with HIV infection and solid organ transplant recipients. Under these conditions, the esophagus becomes susceptible to colonization and infection by fungal, viral, and bacterial pathogens.

Candida species represent the most common cause of infectious esophagitis and are therefore the best-characterized fungal pathogens associated with this condition. *Candida albicans* is the most frequently isolated species. Several studies have demonstrated that mucins such as MUC5AC, MUC5B, and MUC2 inhibit the yeast-to-hyphae transition of *C. albicans*, a critical step for epithelial invasion. In addition, MUC5AC reduces fungal adherence to biotic surfaces, including epithelial cells. Notably, the expression of *C. albicans* adhesin genes, such as ALS1 and ALS3, is downregulated in the presence of mucins. Although *C. albicans* secretes proteases that facilitate tissue invasion, mucins have been shown to suppress the expression of these enzymes, thereby limiting epithelial colonization. Furthermore, interactions between fungal adhesins and secreted mucins promote fungal clearance through mucosal flow mechanisms [57]. Other fungal pathogens implicated in esophageal infections include Aspergillus, Blastomyces, and Cryptococcus species [58].

Viral pathogens may also cause infectious esophagitis, particularly in immunocompromised individuals, and include Cytomegalovirus (CMV), Herpes simplex virus (HSV), Human papillomavirus (HPV), Varicella–zoster virus (VZV), and Epstein–Barr virus (EBV).

The cytopathic effects of enteric and respiratory viruses have been associated with distinct gene expression patterns, including the robust upregulation of mucin genes, for example, MUC2 and MUC5AC in enterovirus infections [59] and MUC19 in Epstein–Barr virus (EBV) infections [60]. Viral antigens are capable of interacting with the promoters of these mucin genes, thereby enhancing their transcription. However, no studies to date have investigated how these mechanisms are manifested in esophageal infections.

Bacterial esophagitis is comparatively rare and typically arises in the context of pre-existing mucosal injury. Reported bacterial pathogens include *Streptococcus viridans*, *Staphylococcus* species, and *Mycobacterium tuberculosis* [61]. Pathogen-associated molecular patterns can activate Toll-like receptor (TLR) signaling pathways in mucosal epithelia, leading to the recruitment and activation of immune cells such as monocytes, eosinophils, basophils, and neutrophils. TLR activation also induces the expression of MUC5AC [62]. Although this mechanism has been primarily characterized in the context of asthma, the esophageal and airway mucosa share key structural and functional features, as both are exposed epithelia, suggesting potential overlap in innate immune responses.

In summary, host-microorganism interactions in the esophageal mucosa are complex and remain incompletely understood. Nevertheless, accumulating evidence indicates that mucins play a regulatory role in these interactions by modulating microbial adhesion, invasion, and clearance. Mucins may contribute to the discrimination between commensal and pathogenic microorganisms, thereby supporting mucosal homeostasis while facilitating the initiation of inflammatory responses when molecular cues signal a risk of pathogenic colonization.

### 4.5. Autoimmune Esophagitis

Esophageal involvement in autoimmune diseases remains poorly characterized in the literature. Evidence regarding molecular changes in the esophageal mucosa—particularly those related to mucin expression and their potential roles in epithelial integrity and immune modulation—is even more limited.

Autoimmune-related esophagitis encompasses a spectrum of conditions, including systemic sclerosis, systemic lupus erythematosus, dermatomyositis/polymyositis, Sjögren’s syndrome, celiac disease, and chronic graft-versus-host disease, each of which may induce esophageal inflammation through distinct immunopathogenic mechanisms. However, these forms of esophagitis are relatively rare, and molecular alterations in the esophageal mucosa have been sparsely investigated. The available findings are summarized below.

Sjögren’s syndrome is an autoimmune disorder that can affect the esophagus through multiple mechanisms. It has been associated with decreased esophageal peristalsis, resulting in dysphagia for solids and retrosternal discomfort. In addition, hyposalivation and xerostomia, in combination with impaired motility, often contribute to the development of GERD. Xerostomia in this context is thought to arise from reduced sulfation of MUC5B and other mucins, whereas hyposalivation has been linked to decreased sialic acid content in the glycosylated regions of mucins. Beyond esophageal manifestations, Sjögren’s syndrome primarily targets exocrine glands, with salivary and lacrimal involvement well documented. Although esophageal gland involvement has not been specifically studied, it is plausible that similar mucin-related abnormalities occur in the esophageal mucosa and associated glands, consistent with reported alterations in MUC1, MUC7, and MUC5B in the salivary glands of affected individuals [63].

A meta-analysis by Niv et al. (2023) reported significant upregulation of MUC5B in the small intestinal mucosa of patients with celiac disease. However, substantial heterogeneity across studies (I^2^ = 80.4%) limits the robustness of this finding, and no data currently exist regarding mucin expression in the esophageal mucosa of patients with celiac disease [64].

Finally, systemic sclerosis [65] and systemic lupus erythematosus [66] are both associated with GERD, which in turn induces alterations in esophageal mucin expression. In these autoimmune disorders, changes in mucin profiles appear to occur indirectly through reflux-mediated mechanisms rather than as a primary pathogenic feature.

### 4.6. Radiation-Induced Esophagitis

Radiation-induced esophagitis is an inflammatory condition of the esophageal mucosa that arises as a consequence of radiotherapy and is commonly observed in patients treated for head and neck or thoracic malignancies, including lung cancer. Clinical symptoms typically develop within 2–3 weeks after the initiation of radiotherapy and may persist for several weeks following treatment completion [67].

In head and neck squamous cell carcinoma (HNSCC) MUC1 overexpression confers radioresistance by inhibiting pro-apoptotic protein expression and impairing apoptosis in response to ionizing radiation [66]. Moreover, MUC1 interacts with SIGLEC-9, promoting the polarization of tumor-associated macrophages (TAMs) toward an immunosuppressive M2 phenotype, thereby contributing to immune evasion and radioresistance. MUC1 also facilitates the nuclear translocation of β-catenin, which suppresses radiotherapy-induced ferroptosis. Notably, these pro-tumorigenic effects are abolished upon inhibition of SIGLEC-9 signaling [68].

In addition, mutations in the MUC19 gene have been associated with poor responsiveness to radiotherapy and reduced overall survival, underscoring its potential utility as a predictive biomarker of treatment resistance. In contrast, increased MUC4 expression has been correlated with more favorable clinical outcomes, suggesting a possible protective or radiosensitizing role [69]. Collectively, these findings support the concept that radiosensitivity and radioresistance are governed by molecular pathways that intersect with inflammatory and immune regulatory mechanisms, in which mucins may serve as important modulators.

### 4.7. Convergent Mechanisms of Mucin Dysregulation in Esophageal Disease

Although drug-induced, caustic, and traumatic esophagitis are well characterized clinically, and their general mechanisms of mucosal injury are well described, there remains a striking paucity of studies investigating molecular alterations in the esophageal epithelium—particularly with respect to mucin expression, composition, and dysregulation—despite the existence of established management strategies and detailed anatomical descriptions for these conditions.

Across diverse forms of esophagitis—including GERD, eosinophilic esophagitis, infectious and autoimmune esophagitis, radiation-induced injury, and premalignant or malignant conditions such as Barrett’s esophagus and esophageal adenocarcinoma—epithelial damage is consistently associated with alterations in mucin expression. Membrane-bound mucins (e.g., MUC1 and MUC4) primarily function as sensors of epithelial stress and modulators of inflammatory signaling, whereas secreted mucins (e.g., MUC2, MUC5AC, MUC5B, and MUC6) predominantly contribute to mucus barrier formation and epithelial protection. Inflammatory stimuli—including cytokines such as IL-1β, IL-6, IL-8, and TNF-α, as well as activation of NF-κB and STAT signaling pathways—are tightly linked to mucin dysregulation, highlighting a bidirectional relationship in which mucins both respond to inflammatory cues and actively modulate inflammatory processes. This dysregulation typically involves the upregulation of injury-associated mucins alongside the downregulation of protective mucins, ultimately compromising epithelial defenses and perpetuating mucosal inflammation.

## 5. Discussion

Esophageal inflammation is a common feature of chronic disorders such as GERD, EoE, Barrett’s esophagus, and esophageal cancer. Mucins, a family of highly glycosylated epithelial glycoproteins, are key regulators of the mucosal barrier and inflammatory responses. Alterations in mucin expression, localization, and glycosylation are frequently observed under inflammatory conditions and may contribute to sustained epithelial injury and disease chronicity. A deeper understanding of the relationship between mucins and inflammation may therefore facilitate the identification of novel therapeutic targets aimed at limiting chronic inflammation and preventing disease progression.

Most published studies addressing mucin biology fall into two major research contexts: host-microorganism interactions and tissue malignancy. In the first context, research has focused on mucin-mediated defense mechanisms against pathogenic microorganisms, as well as on the nutritional support and spatial regulation of commensal microbial populations. These processes are essential for maintaining physical separation between the microbiota and the epithelial surface, thereby preventing inappropriate colonization. Numerous studies have explored these interactions in organs such as the stomach—particularly in relation to *Helicobacter pylori*—and the colon, where mucins play a central role in shaping host-microbe co-evolution. Depending on the context, mucin-mediated interactions may promote mutualistic outcomes, such as the establishment of a stable mucosal microbiota, or contribute to pathological states, including pathogenic invasion. In the second context, an extensive body of literature has examined alterations in mucin subtype expression, subcellular localization, glycosylation patterns, oligomerization, and proteolytic processing during malignant transformation and tumor progression across multiple gastrointestinal organs and other mucosal tissues. These studies have underscored the dynamic regulation of mucins during carcinogenesis.

However, beyond inflammation-associated carcinogenesis, relatively little is known about purely inflammatory mechanisms in which mucins are directly or indirectly involved. Data addressing mucin function in non-malignant inflammatory conditions remain scarce, particularly in the esophagus. Although emerging evidence highlights mucin dysregulation in specific forms of esophagitis, further research is required to define the precise mechanistic roles of mucins in these settings.

Structurally, mucins possess tandem repeat domains within their core protein, ranging from 5 to 500 repeats, each containing 5–100 potential glycosylation sites. This remarkable structural complexity enables the recognition and binding of a wide array of ligands through selective ligand–receptor interactions. As a result, mucins fulfill diverse functions essential for epithelial homeostasis, including microbial recognition, initiation of immune responses against pathogens, and formation of a gel-like mucus layer composed of polymerized mucins and associated molecules [3]. This mucus gel selectively permits the passage of certain substances while restricting the access of others to the epithelial surface, thereby facilitating clearance through the gastrointestinal tract. In addition, the extracellular domains of transmembrane mucins can be released through regulated proteolytic cleavage, allowing them to integrate into the mucus layer and extend their functional range [70].

Beyond barrier formation, specific structural motifs confer mucins with immunomodulatory properties. For example, the presence of EGF-like domains in mucins such as MUC4, which interacts with HER2, or MUC12, which can release its EGF-like domain, enables these proteins to participate in cytokine-like signaling and immune regulation. Membrane-associated mucins have also been detected in activated lymphocytes, and several mucins are capable of binding leukocytes through interactions with Siglecs and selectins [71,72,73,74,75]. Selectin-mucin interactions are particularly relevant for leukocyte adhesion, extravasation, and motility during inflammatory responses [76].

During inflammation, mucins are not only expressed by epithelial and immune cells but also function as reservoirs for cytokines. Human interleukins—including IL-1, IL-4, IL-6, and IL-7—can bind to mucins through lectin-like interactions. Moreover, cytokines have been detected within the mucus of respiratory and gastrointestinal epithelia, where they contribute to mucus hypersecretion and selective induction of specific mucins as part of the inflammatory response [77,78,79,80].

A distinct and well-established body of research has focused on characterizing mucin expression patterns under physiological and pathological conditions, often through comparative analyses of healthy and diseased tissues. Within this framework, mucins have emerged as valuable diagnostic and prognostic biomarkers, particularly in cancer, owing to their tissue-specific expression and localization. However, many of these studies provide limited mechanistic insight into how altered mucin expression contributes to disease initiation or progression. In particular, the molecular pathways through which mucins modulate or perpetuate inflammation—especially in tumor-associated contexts—remain insufficiently explored. Despite these limitations, mucins continue to represent promising candidates for clinical stratification and are increasingly recognized as potential therapeutic targets not only in oncology but also in inflammatory diseases.

An additional consideration is that the esophageal epithelium has traditionally been regarded as a non-mucus-secreting tissue. Nonetheless, the esophagus contains submucosal glands and, together with the epithelial layer, contributes to the luminal release of mucins and other bioactive molecules. Unlike other regions of the gastrointestinal tract, the esophageal epithelium lacks specialized secretory cells such as goblet cells, conferring unique functional characteristics that reflect its specific physiological role.

Importantly, under normal physiological conditions, swallowed saliva continuously enters the esophageal lumen. Consequently, some mucins exerting functional roles in the esophagus may originate not from local secretion but from proximal salivary glands. This persistent exposure to salivary components may confound interpretations that attribute mucin-related functions exclusively to esophageal epithelial sources. For this reason, mucins such as MUC7, although not synthesized locally, warrant investigation due to their potential contribution to mucosal protection and modulation of luminal interactions. Furthermore, the short half-life of MUC7 may hinder its detection in routine analyses, potentially leading to underestimation of its presence and functional relevance. Accordingly, salivary mucins have been included and considered in the present review.

The effect of dietary behaviors on barrier integrity and mucin structure is a topic of growing interest. The interplay between diet, microbiota, mucus, and the intestinal immune system has begun to be unveiled, and along with it, the impact that different dietary patterns have on intestinal homeostasis and the risk of developing inflammatory diseases. At the esophageal level, various chemical irritants—including alcohol, caffeine, and highly spicy or acidic foods (such as citrus and tomato)—can alter the composition of the mucus layer, diminishing its protective capacity against acid and digestive enzymes [81,82]. Diets rich in saturated fats and fried foods relax the lower esophageal sphincter and delay gastric emptying, facilitating reflux and increasing acid exposure time, thereby altering the interaction between the mucus and the epithelium. In addition, diets low in fiber and high in fat and sugars have been associated with alterations in microbiota composition/functionality and the subsequent development of chronic diseases such as food allergies [83], inflammatory bowel disease, and metabolic disease [84]. On the contrary, diets high in fiber and low in fat and sugars have been shown to have a beneficial effect on intestinal health [85]: dietary components, such as fermentable fiber and other polysaccharides, can influence mucin O-glycosylation profiles and glycan-processing pathways, thereby modulating mucin structural properties through host–microbiota metabolic interactions in other regions of the gastrointestinal tract beyond the esophagus [86]. As a result, they are able to promote the growth of beneficial bacteria, improve mucus barrier function and immune tolerance, while inhibiting pro-inflammatory responses and their downstream effects [84,87].

In conclusion, a more comprehensive understanding of mucin–inflammation interactions is essential for advancing our knowledge of esophageal pathophysiology. Such insight may ultimately enable the development of targeted therapeutic strategies for mucosal inflammatory conditions such as esophagitis, in which mucin dysregulation likely plays a contributory pathogenic role.

## Figures and Tables

**Table 1:** Expression and inflammatory relevance of mucins in the esophagus.

Mucin Type	Associated Inflammatory Disorders	Basal Esophageal Expression	Inflammation-Related Findings
**MUC1**	Multiple cancers	Low	Upregulated by pro-inflammatory cytokines (IFN-γ, TNF-α, IL-1, IL-6); promotes chronic inflammation; modulates TLR signaling and immune cell recruitment; exhibits aberrant glycosylation under inflammatory conditions.
**MUC2**	IBD, Barrett’s esophagus, intestinal metaplasia	Absent	NF-κB–regulated; downregulated in IBD; depletion compromises mucus barrier integrity, exacerbating inflammation; ectopic expression in Barrett’s esophagus associated with metaplastic responses.
**MUC3**	IBD, GERD, Barrett’s esophagus, esophageal cancer	Absent	Modulates inflammatory responses and mucin–bacteria interactions; correlates with IL-6 levels; increased expression in GERD; associated with inflammation in IBD and intestinal metaplasia.
**MUC4**	EoE, Barrett’s esophagus, esophageal cancer, Crohn’s disease, cystic fibrosis, COPD	Low	Upregulated in EoE and Barrett’s esophagus; elevated in inflamed EoE mucosa; regulated by IL-1 and IL-9 via JAK3/STAT6 signaling in respiratory epithelium; altered in Crohn’s disease, cystic fibrosis, and COPD; inflammatory stimuli affect transcription and alternative splicing.
**MUC5B**	Respiratory diseases, EoE	Low (submucosal glands)	Binds TLR4 and interacts with Siglec-8; induces immune cell activation and sustains inflammation in EoE; exhibits pro-inflammatory roles in salivary glands; expression regulated by cytokines and epigenetic mechanisms.
**MUC5AC**	Barrett’s esophagus, esophageal cancer	Absent	Induced by IL-13 during allergic inflammation; associated with eosinophilic signaling in EoE; co-expressed with MUC5B in airway inflammation; involved in gastric and esophageal mucosal remodeling under inflammatory conditions.
**MUC6**	GERD, Barrett’s esophagus, intestinal metaplasia, Crohn’s disease	Absent	Induced by pro-inflammatory cytokines (IL-1β, IL-6, TNF-α) and LPS; upregulated in Crohn’s disease; aberrantly expressed in GERD and Barrett’s esophagus as part of a metaplastic response.
**MUC7**	Recurrent aphthous stomatitis, pyelonephritis	Not expressed (saliva-secreted)	Functions as a chemoattractant for pro-inflammatory mediators; increased expression and sulfation reported in rheumatoid arthritis; altered during infection-related inflammation; glycosylation changes observed in recurrent aphthous stomatitis; increased levels in pyelonephritis.
**MUC13**	Esophageal cancer	Absent	Downregulated during enterobacterial infection; implicated in glycosylation-dependent inflammatory signaling; modulated by microbial components.
**MUC15**	Esophageal cancer, respiratory infections	Low	Upregulated during respiratory syncytial virus and influenza virus infection; may exert immunomodulatory functions during viral responses.
**MUC16**	Esophageal cancer, EoE	Low	Induced by IL-1, TNF-α, and LPS in vitro; associated with inflammatory and immune pathways, including heart failure–related inflammation; genetic variants linked to EoE.
**MUC19**	Esophageal cancer, murine colitis	Not expressed (saliva-secreted)	Deficiency exacerbates colitis severity in mouse models.
**MUC20**	Multiple cancers, respiratory infections	Low	Induced by respiratory syncytial virus infection; involved in inflammatory responses in respiratory epithelium.
**MUC21**	Esophageal cancer, respiratory infections	Moderate	Upregulated during respiratory infections; potentially involved in epithelial inflammatory responses.
**MUC22**	Respiratory infections, cancer	Low/variable	Induced by respiratory syncytial virus infection; polymorphisms associated with inflammatory disease susceptibility and protective traits during COVID-19 recovery.

MUC: Mucin; IBD—Inflammatory Bowel Disease; GERD—Gastroesophageal Reflux Disease; EoE—Eosinophilic Esophagitis; COPD—Chronic Obstructive Pulmonary Disease; IFN-γ: Interferon gamma; TNF-α: Tumor Necrosis Factor alpha; IL-1: Interleukin-1; IL-6: Interleukin-6; TLR: Toll-Like Receptor; NF-κB: Nuclear Factor kappa B; IL-9: Interleukin-9; JAK3/STAT6: Janus Kinase 3/Signal Transducer and Activator of Transcription 6; TLR4: Toll-Like Receptor 4; IL-13: Interleukin-13; MUC5B: Mucin 5B; IL-1β: Interleukin-1 beta; LPS: Lipopolysaccharide; COVID-19: Coronavirus Disease 2019.

## Data Availability

No new data were created or analyzed in this study. Data sharing is not applicable to this article.

## Abbreviations

The following abbreviations are used in this manuscript:

MUC	Mucin
GERD	Gastroesophageal Reflux Disease
EoE	Eosinophilic Esophagitis
IFN-γ	Interferon gamma
IL	Interleukin
TNF-α	Tumor Necrosis Factor alpha
IBD	Inflammatory Bowel Disease
TLR	Toll-Like Receptor
LPS	Lipopolysaccharide
COPD	Chronic Obstructive Pulmonary Disease
NF-κB	Nuclear Factor kappa B
NERD	Non-Erosive Reflux Disease
PPIs	Proton Pump Inhibitors
LGD	Low-Grade Dysplasia
HGD	High-Grade Dysplasia
PER-1	Period Circadian Regulator 1
iNOS	Inducible Nitric Oxide Synthase
GZMA	Granzyme A
NKG2D	Natural Killer Group 2 Member D
KLRK1	Killer Cell Lectin-Like Receptor K1
MICB	MHC Class I Polypeptide-Related Sequence B
SFED	Six-Food Elimination Diet
ALS	Agglutinin-Like Sequence
CMV	Cytomegalovirus
HSV	Herpes Simplex Virus
HPV	Human Papillomavirus
VZV	Varicella-Zoster Virus
EBV	Epstein–Barr Virus
ESCC	Esophageal Squamous Cell Carcinoma
SIGLEC	Sialic Acid-Binding Immunoglobulin-Like Lectin
STAT	Signal Transducer and Activator of Transcription
EGF	Epidermal Growth Factor
